# Dr. Basil Norris

**Published:** 1895-12

**Authors:** 


### Obituary.

DR. BASIL NORRIS, Colonel U. S. Army, retired, died at San Francisco, Cal., November 11, 1895, aged 67 years. Dr. Norris graduated at the University of Maryland in 1849 and entered the army as assistant surgeon in 1852. He served in Texas, Utah and New Mexico until 1862, when he was promoted to surgeon and appointed inspector of hospitals in October of the same year. When Burnside organised the Army of the Potomac into grand divisions, in November, 1862, Dr. Norris was appointed medical director of the left grand division, serving on the staff of General Franklin. When the grand divisions were abolished he was assigned to duty as attending surgeon at Washington. The writer enjoyed Dr. Norris's friendship during his service in the field with the Army of the Potomac, and met him often during his service in Washington. He was a most charming man, courteous in his demeanor, considerate of his subordinates, prompt in the discharge of his duty, skilful in his profession, and an ornament to his corps. He died deeply lamented both in military and civil life.

